# Bis[2-(methyl­amino)­troponato]copper(II)

**DOI:** 10.1107/S1600536810043503

**Published:** 2010-11-06

**Authors:** Gideon Steyl, Theunis J. Muller, Andreas Roodt

**Affiliations:** a Department of Chemistry, University of the Free State, PO Box 339, Bloemfontein 9300, South Africa

## Abstract

In the title compound, [Cu(C_8_H_8_NO)_2_], a strictly square-planar geometry about the Cu^II^ metal atom is observed. Substitution of an O atom with a methyl-functionalized N atom does not significantly alter the bond distances and angles in the copper(II) complex when compared with a similar bis­(troponato)copper(II) complex. π–π stacking is observed between the tropolone rings, with inter­planar distances of 3.5039 (16) and 3.2933 (15) Å, respectively. Additional stabilisation of the structure is accomplished through C—H⋯O hydrogen-bonding interactions.

## Related literature

For related literature on values of bond lengths and angles, see: Zhang *et al.* (2008 ▶); Hill & Steyl (2008 ▶); Kristiansson (2002 ▶). For similar structures, see: Liang *et al.* (2001 ▶). For other related structures, see: Starikova & Shugam (1969 ▶); Byrn *et al.* (1993 ▶); Park & Marshall (2005 ▶); Dessy & Fares (1979 ▶); Baidina *et al.* (2004 ▶). For the synthesis of the title compound, see: Roesky & Burgstein (1999 ▶); Claramunt *et al.* (2004 ▶). For background and the use of the title compound, see: Roesky (2000 ▶); Nepveu *et al.* (1993 ▶); Crous *et al.* (2005 ▶); Roodt *et al.* (2003 ▶); Steyl (2005 ▶); Steyl *et al.* (2001 ▶).
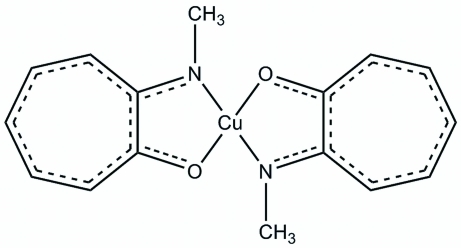


## Experimental

### 

#### Crystal data

[Cu(C_8_H_8_NO)_2_]
*M* *_r_* = 331.85
Monoclinic, 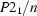

*a* = 6.7541 (9) Å
*b* = 9.1599 (12) Å
*c* = 22.084 (3) Å
β = 92.108 (5)°
*V* = 1365.3 (3) Å^3^
*Z* = 4
Mo *K*α radiation
μ = 1.61 mm^−1^
*T* = 100 K
0.34 × 0.32 × 0.17 mm

#### Data collection

Bruker APEXII CCD area-detector diffractometer
Absorption correction: multi-scan (*SADABS*; Bruker, 2004 ▶) *T*_min_ = 0.583, *T*_max_ = 0.760
21665 measured reflections
2985 independent reflections
2804 reflections with *I* > 2σ(*I*)
*R*_int_ = 0.032

#### Refinement

*R*[*F* ^2^ > 2σ(*F* ^2^)] = 0.022
*wR*(*F* ^2^) = 0.066
*S* = 1.06
2985 reflections
190 parameters
H-atom parameters constrained
Δρ_max_ = 0.36 e Å^−3^
Δρ_min_ = −0.38 e Å^−3^

### 

Data collection: *APEX2* (Bruker, 2005 ▶); cell refinement: *SAINT-Plus* (Bruker, 2004 ▶); data reduction: *SAINT-Plus*; program(s) used to solve structure: *SHELXS97* (Sheldrick, 2008 ▶); program(s) used to refine structure: *SHELXL97* (Sheldrick, 2008 ▶); molecular graphics: *DIAMOND* (Brandenberg & Putz, 2004 ▶); software used to prepare material for publication: *WinGX* (Farrugia, 1999 ▶).

## Supplementary Material

Crystal structure: contains datablocks global, I. DOI: 10.1107/S1600536810043503/pk2271sup1.cif

Structure factors: contains datablocks I. DOI: 10.1107/S1600536810043503/pk2271Isup2.hkl

Additional supplementary materials:  crystallographic information; 3D view; checkCIF report

## Figures and Tables

**Table 1:** Hydrogen-bond geometry (Å, °)

*D*—H⋯*A*	*D*—H	H⋯*A*	*D*⋯*A*	*D*—H⋯*A*
C18—H18*A*⋯O2	0.98	2.47	3.1222 (18)	124
C28—H28*A*⋯O1	0.98	2.41	3.0715 (18)	124

**Fig. 1.**
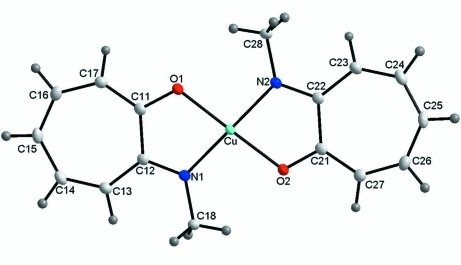
Representation of the title compound, showing the numbering scheme and displacement ellipsoids (50% probability).

## supplementary crystallographic information

### Comment

Complexes containing tropolonato type derivatives have steadily increased over the last few decades (Roesky, 2000), with most of the work having involved the first and second row transition elements. This attention has been to a large extent due to the medical application of tropolone in radio-pharmaceuticals (Nepveu *et al.*, 1993) and catalyst precursors (Crous *et al.*, 2005; Roodt *et al.*, 2003). Functionalization of the tropolonato backbone (seven-membered ring) has also been investigated with a range of Rh^I^ and Pd^II^ complexes reported to date (Steyl *et al.*, 2001; Steyl, 2005).

Heteroatom substitution of the tropolonato moiety has also been reported, most notably the O atoms are replaced with functionalized amino groups, resulting in either the 2- (aminotropone) or 1,2- (aminotropoimine) compounds (Roesky & Burgstein, 1999; Claramunt *et al.*, 2004). Thus, a host of amino and anilino derivatives of tropolone have been reported (Roesky & Burgstein, 1999; Roesky, 2000; Claramunt *et al.*, 2004). The addition of electron-donating or -withdrawing moieties to the N atom can significantly increase the application of these compounds in coordination chemistry. The most interesting observation concerning the Cu^II^ metal centres in general is the difference in the coordination behaviour of the bidentate-*O*,*O* donor atoms compared to the *N*,*N* donor atom complexes; the geometrical conformation changes from a square planar (Starikova & Shugam, 1969; Byrn *et al.*, 1993) to a tetrahedral geometry (Park & Marshall, 2005; Dessy & Fares, 1979), respectively. The *N*-methylaminoacetylacetonato derivative have been reported (Baidina *et al.*, 2004) as a distorted tetrahedral complex.

In an effort to further investigate these types of complexes, the crystal structure of [Cu(TropNMe)_2_] is presented.

The copper(II) ion is coordinated by two TropNMe ligands in a square-planar geometry (Fig 1). The Cu^2+^ ion chelates the TropNMe ligand *via* N1 and O1 to form a five-membered ring. The N1—O1—Cu—N2—O2 moiety is strictly planar. The ligand planes form angles of 6.54 (1)° and 3.12 (1)°, respectively with the 5-membered ring. This compares with literature (Hill & Steyl, 2008). π–π stacking between C11–C17 and C11–C17^i^ (-*x*, 1 - *y*, -*z*) as well as C11–C17 and C21–C27^ii^ (-*x*, 2 - *y*, -*z*) with intercentroid distances of 4.1483 (4) Å and 3.7827 (4) Å respectively as defined by the seven-membered ring system. Cu—O1 and Cu—O2 bond distances is 1.9313 (2) Å and 1.9386 (2) Å, respectively. This correlates well with literature (Zhang *et al.*, 2008). The Cu—N1 and Cu—N2 bond distances is 1.9276 (2) Å and 1.9291 (2) Å respectively. The O1—Cu—N1 and O2—Cu—N2 angles is 82.292 (4)° and 82.090 (4)° respectively. This correlates well with literature (Kristiansson 2002). The N1—Cu—N2 angle is 175.769 (5)° this is smaller than the O1—Cu—O2 angle, which is 179.229 (6)°. This is in accordance with literature (Liang *et al.*, 2001). The title compound is further stabilized by weak intramolecular C15—H15*A*···O1 and C16—H16*A*···O2 hydrogen interactions.

### Experimental

Synthesis of 2-(*N*-methylamino)tropone (HTropNMe) was done according to the literature procedure (Roesky & Burgstein, 1999; Claramunt *et al.*, 2004), while all other starting materials were obtained from commercially available sources. Cu(NO_3_)_2_ (100 mg, 0.44 mmol) was dissolved in CHCl_3_ or MeOH and refluxed with HTropNMe for 30 min. The product was filtered from the cold solution and dried in air for 24 h. Rhombic dark-green crystals suitable for X-ray diffraction was obtained from a chloroform/ether (1:1, 10 ml) mixture after 2 d. (Yield: 117 mg, 70%) ^1^H NMR (300 MHz, CDCl_3_, 25°C) 7.32 (m, 2H), 7.12 (d, 1H), 6.95 (d, 1H), 6.81 (t, 1H), 1.56 (s, 3H)

### Refinement

All H atoms were positioned geometrically and allowed to ride on their parent atoms, with *U*_iso_(H) = 1.2U_eq_(parent) of the parent atom with a C—H distance of 0.93. The methyl H atoms were placed in geometrically idealized positions and constrained to ride on the parent atoms with *U*_iso_(H) = 1.5*U*_eq_(C) and at a distance of 0.96 Å.

### Crystal data

**Table:**

[Cu(C_8_H_8_NO)_2_]	*F*(000) = 684
*M**_r_* = 331.85	*D*_x_ = 1.614 Mg m^−^^3^
Monoclinic, *P*2_1_/*n*	Mo *K*α radiation, λ = 0.71073 Å
Hall symbol: -P 2yn	Cell parameters from 6754 reflections
*a* = 6.7541 (9) Å	θ = 2.4–28.2°
*b* = 9.1599 (12) Å	µ = 1.61 mm^−^^1^
*c* = 22.084 (3) Å	*T* = 100 K
β = 92.108 (5)°	Cuboid, black
*V* = 1365.3 (3) Å^3^	0.34 × 0.32 × 0.17 mm
*Z* = 4	

### Data collection

**Table:**

Bruker APEXII CCD area-detector diffractometer	2985 independent reflections
graphite	2804 reflections with *I* > 2σ(*I*)
Detector resolution: 8.5 pixels mm^-1^	*R*_int_ = 0.032
φ and ω scans	θ_max_ = 27.0°, θ_min_ = 2.4°
Absorption correction: multi-scan (*SADABS*; Bruker, 2004)	*h* = −8→8
*T*_min_ = 0.583, *T*_max_ = 0.760	*k* = −10→11
21665 measured reflections	*l* = −28→28

### Refinement

**Table:**

Refinement on *F*^2^	Primary atom site location: structure-invariant direct methods
Least-squares matrix: full	Secondary atom site location: difference Fourier map
*R*[*F*^2^ > 2σ(*F*^2^)] = 0.022	Hydrogen site location: inferred from neighbouring sites
*wR*(*F*^2^) = 0.066	H-atom parameters constrained
*S* = 1.06	*w* = 1/[σ^2^(*F*_o_^2^) + (0.0363*P*)^2^ + 0.8483*P*] where *P* = (*F*_o_^2^ + 2*F*_c_^2^)/3
2985 reflections	(Δ/σ)_max_ < 0.001
190 parameters	Δρ_max_ = 0.36 e Å^−^^3^
0 restraints	Δρ_min_ = −0.38 e Å^−^^3^

### Special details

**Table:**

Experimental. First 80 frames repeated after collection for monitoring possible decay.
Geometry. All s.u.'s (except the s.u. in the dihedral angle between two l.s. planes) are estimated using the full covariance matrix. The cell s.u.'s are taken into account individually in the estimation of s.u.'s in distances, angles and torsion angles; correlations between s.u.'s in cell parameters are only used when they are defined by crystal symmetry. An approximate (isotropic) treatment of cell s.u.'s is used for estimating s.u.'s involving l.s. planes.
Refinement. Refinement of *F*^2^ against ALL reflections. The weighted *R*-factor *wR* and goodness of fit *S* are based on *F*^2^, conventional *R*-factors *R* are based on *F*, with *F* set to zero for negative *F*^2^. The threshold expression of *F*^2^ > 2σ(*F*^2^) is used only for calculating *R*-factors(gt) *etc*. and is not relevant to the choice of reflections for refinement. *R*-factors based on *F*^2^ are statistically about twice as large as those based on *F*, and *R*-factors based on ALL data will be even larger.

### Fractional atomic coordinates and isotropic or equivalent isotropic displacement parameters (Å^2^)

**Table:**

	*x*	*y*	*z*	*U*_iso_*/*U*_eq_	
C11	0.0756 (2)	0.73761 (16)	−0.03802 (6)	0.0123 (3)	
C12	−0.1170 (2)	0.68077 (16)	−0.01916 (6)	0.0117 (3)	
C13	−0.2292 (2)	0.57152 (18)	−0.05113 (7)	0.0152 (3)	
H13	−0.3515	0.5479	−0.0336	0.018*	
C14	−0.1913 (2)	0.49449 (18)	−0.10280 (7)	0.0172 (3)	
H14	−0.2907	0.4259	−0.1147	0.021*	
C15	−0.0299 (2)	0.50069 (18)	−0.14071 (7)	0.0171 (3)	
H15	−0.0326	0.4366	−0.1746	0.02*	
C16	0.1324 (2)	0.59075 (18)	−0.13361 (7)	0.0157 (3)	
H16	0.2282	0.5813	−0.1638	0.019*	
C17	0.1773 (2)	0.69413 (17)	−0.08858 (7)	0.0141 (3)	
H17	0.2993	0.7436	−0.0934	0.017*	
C18	−0.3658 (2)	0.70243 (17)	0.05564 (6)	0.0136 (3)	
H18A	−0.3873	0.7593	0.0924	0.02*	
H18B	−0.364	0.5982	0.0656	0.02*	
H18C	−0.4732	0.7221	0.0257	0.02*	
C21	−0.0559 (2)	1.02279 (16)	0.17475 (6)	0.0119 (3)	
C22	0.1447 (2)	1.06912 (16)	0.15765 (6)	0.0113 (3)	
C23	0.2695 (2)	1.16602 (17)	0.19214 (7)	0.0145 (3)	
H23	0.3988	1.1773	0.1773	0.017*	
C24	0.2340 (2)	1.24600 (17)	0.24341 (7)	0.0172 (3)	
H24	0.3422	1.3042	0.2578	0.021*	
C25	0.0645 (2)	1.25541 (17)	0.27773 (7)	0.0176 (3)	
H25	0.0693	1.3219	0.3108	0.021*	
C26	−0.1089 (2)	1.17780 (18)	0.26841 (7)	0.0167 (3)	
H26	−0.2094	1.1981	0.2962	0.02*	
C27	−0.1587 (2)	1.07348 (18)	0.22402 (7)	0.0146 (3)	
H27	−0.2848	1.0296	0.2283	0.018*	
C28	0.3903 (2)	1.04390 (17)	0.08191 (7)	0.0136 (3)	
H28A	0.4064	0.9896	0.0442	0.02*	
H28B	0.4966	1.0171	0.1112	0.02*	
H28C	0.3965	1.1489	0.0737	0.02*	
N1	−0.17729 (18)	0.74362 (14)	0.03074 (5)	0.0117 (2)	
N2	0.19904 (18)	1.00838 (14)	0.10669 (5)	0.0117 (2)	
O1	0.15352 (16)	0.83831 (13)	−0.00342 (5)	0.0158 (2)	
O2	−0.14060 (15)	0.92738 (13)	0.13907 (5)	0.0142 (2)	
Cu	0.00529 (2)	0.881953 (19)	0.067482 (7)	0.01073 (8)	

### Atomic displacement parameters (Å^2^)

**Table:**

	*U*^11^	*U*^22^	*U*^33^	*U*^12^	*U*^13^	*U*^23^
C11	0.0124 (7)	0.0115 (7)	0.0126 (6)	0.0001 (5)	−0.0022 (5)	0.0015 (5)
C12	0.0118 (7)	0.0108 (7)	0.0122 (6)	0.0004 (5)	−0.0013 (5)	0.0018 (5)
C13	0.0146 (7)	0.0149 (8)	0.0161 (7)	−0.0032 (6)	0.0009 (5)	−0.0008 (6)
C14	0.0190 (7)	0.0146 (8)	0.0178 (7)	−0.0037 (6)	−0.0027 (6)	−0.0030 (6)
C15	0.0222 (8)	0.0142 (8)	0.0147 (7)	0.0012 (6)	−0.0007 (6)	−0.0048 (6)
C16	0.0183 (7)	0.0160 (8)	0.0128 (7)	0.0034 (6)	0.0016 (6)	−0.0005 (6)
C17	0.0137 (7)	0.0136 (8)	0.0150 (7)	−0.0007 (6)	0.0004 (5)	0.0005 (5)
C18	0.0122 (7)	0.0146 (8)	0.0142 (7)	−0.0020 (6)	0.0019 (5)	−0.0006 (5)
C21	0.0135 (7)	0.0103 (7)	0.0116 (6)	0.0000 (5)	−0.0024 (5)	0.0028 (5)
C22	0.0120 (7)	0.0094 (7)	0.0123 (6)	0.0013 (5)	−0.0016 (5)	0.0024 (5)
C23	0.0134 (7)	0.0143 (8)	0.0158 (7)	−0.0009 (6)	−0.0013 (5)	0.0000 (6)
C24	0.0210 (8)	0.0134 (8)	0.0166 (7)	−0.0028 (6)	−0.0048 (6)	−0.0014 (6)
C25	0.0274 (8)	0.0133 (8)	0.0120 (7)	−0.0008 (6)	−0.0006 (6)	−0.0026 (5)
C26	0.0224 (8)	0.0162 (8)	0.0118 (7)	0.0015 (6)	0.0030 (6)	0.0001 (6)
C27	0.0160 (7)	0.0150 (8)	0.0128 (7)	−0.0010 (6)	0.0006 (5)	0.0017 (6)
C28	0.0106 (7)	0.0151 (8)	0.0152 (7)	−0.0013 (5)	0.0006 (5)	−0.0007 (5)
N1	0.0115 (6)	0.0115 (6)	0.0122 (6)	−0.0012 (5)	0.0000 (4)	0.0008 (5)
N2	0.0114 (6)	0.0110 (6)	0.0127 (6)	−0.0007 (5)	−0.0011 (4)	−0.0003 (5)
O1	0.0145 (5)	0.0185 (6)	0.0143 (5)	−0.0058 (4)	0.0019 (4)	−0.0048 (4)
O2	0.0137 (5)	0.0167 (6)	0.0123 (5)	−0.0035 (4)	0.0008 (4)	−0.0027 (4)
Cu	0.01051 (11)	0.01176 (12)	0.00990 (11)	−0.00228 (6)	−0.00007 (7)	−0.00182 (6)

### Geometric parameters (Å, °)

**Table:**

C11—O1	1.2970 (18)	C21—C22	1.482 (2)
C11—C17	1.390 (2)	C22—N2	1.3196 (19)
C11—C12	1.475 (2)	C22—C23	1.425 (2)
C12—N1	1.3210 (19)	C23—C24	1.377 (2)
C12—C13	1.426 (2)	C23—H23	0.95
C13—C14	1.374 (2)	C24—C25	1.399 (2)
C13—H13	0.95	C24—H24	0.95
C14—C15	1.400 (2)	C25—C26	1.379 (2)
C14—H14	0.95	C25—H25	0.95
C15—C16	1.376 (2)	C26—C27	1.401 (2)
C15—H15	0.95	C26—H26	0.95
C16—C17	1.398 (2)	C27—H27	0.95
C16—H16	0.95	C28—N2	1.4582 (18)
C17—H17	0.95	C28—H28A	0.98
C18—N1	1.4551 (18)	C28—H28B	0.98
C18—H18A	0.98	C28—H28C	0.98
C18—H18B	0.98	N1—Cu	1.9263 (12)
C18—H18C	0.98	N2—Cu	1.9287 (12)
C21—O2	1.2958 (18)	O1—Cu	1.9312 (11)
C21—C27	1.392 (2)	O2—Cu	1.9385 (11)
			
O1—C11—C17	118.36 (13)	C24—C23—H23	114.6
O1—C11—C12	115.32 (13)	C22—C23—H23	114.6
C17—C11—C12	126.32 (14)	C23—C24—C25	130.46 (15)
N1—C12—C13	122.96 (13)	C23—C24—H24	114.8
N1—C12—C11	112.57 (13)	C25—C24—H24	114.8
C13—C12—C11	124.46 (13)	C26—C25—C24	126.51 (15)
C14—C13—C12	131.26 (14)	C26—C25—H25	116.7
C14—C13—H13	114.4	C24—C25—H25	116.7
C12—C13—H13	114.4	C25—C26—C27	129.55 (15)
C13—C14—C15	130.47 (15)	C25—C26—H26	115.2
C13—C14—H14	114.8	C27—C26—H26	115.2
C15—C14—H14	114.8	C21—C27—C26	131.35 (15)
C16—C15—C14	126.19 (15)	C21—C27—H27	114.3
C16—C15—H15	116.9	C26—C27—H27	114.3
C14—C15—H15	116.9	N2—C28—H28A	109.5
C15—C16—C17	129.65 (15)	N2—C28—H28B	109.5
C15—C16—H16	115.2	H28A—C28—H28B	109.5
C17—C16—H16	115.2	N2—C28—H28C	109.5
C11—C17—C16	131.64 (15)	H28A—C28—H28C	109.5
C11—C17—H17	114.2	H28B—C28—H28C	109.5
C16—C17—H17	114.2	C12—N1—C18	120.21 (12)
N1—C18—H18A	109.5	C12—N1—Cu	115.20 (10)
N1—C18—H18B	109.5	C18—N1—Cu	124.54 (10)
H18A—C18—H18B	109.5	C22—N2—C28	120.26 (12)
N1—C18—H18C	109.5	C22—N2—Cu	115.52 (10)
H18A—C18—H18C	109.5	C28—N2—Cu	124.15 (10)
H18B—C18—H18C	109.5	C11—O1—Cu	114.43 (9)
O2—C21—C27	118.58 (13)	C21—O2—Cu	114.48 (9)
O2—C21—C22	115.20 (13)	N1—Cu—N2	175.77 (5)
C27—C21—C22	126.21 (14)	N1—Cu—O1	82.26 (5)
N2—C22—C23	122.75 (13)	N2—Cu—O1	97.17 (5)
N2—C22—C21	112.46 (13)	N1—Cu—O2	98.51 (5)
C23—C22—C21	124.78 (13)	N2—Cu—O2	82.06 (5)
C24—C23—C22	130.81 (14)	O1—Cu—O2	179.23 (4)
			
O1—C11—C12—N1	−0.08 (18)	C13—C12—N1—C18	0.0 (2)
C17—C11—C12—N1	−179.70 (14)	C11—C12—N1—C18	179.21 (12)
O1—C11—C12—C13	179.09 (14)	C13—C12—N1—Cu	177.49 (11)
C17—C11—C12—C13	−0.5 (2)	C11—C12—N1—Cu	−3.32 (16)
N1—C12—C13—C14	−179.73 (16)	C23—C22—N2—C28	−2.6 (2)
C11—C12—C13—C14	1.2 (3)	C21—C22—N2—C28	178.48 (12)
C12—C13—C14—C15	−0.7 (3)	C23—C22—N2—Cu	−179.61 (11)
C13—C14—C15—C16	−0.3 (3)	C21—C22—N2—Cu	1.49 (16)
C14—C15—C16—C17	0.5 (3)	C17—C11—O1—Cu	−176.94 (11)
O1—C11—C17—C16	−179.83 (16)	C12—C11—O1—Cu	3.41 (16)
C12—C11—C17—C16	−0.2 (3)	C27—C21—O2—Cu	173.51 (11)
C15—C16—C17—C11	0.1 (3)	C22—C21—O2—Cu	−5.60 (16)
O2—C21—C22—N2	2.74 (18)	C12—N1—Cu—O1	4.09 (10)
C27—C21—C22—N2	−176.28 (14)	C18—N1—Cu—O1	−178.57 (12)
O2—C21—C22—C23	−176.13 (14)	C12—N1—Cu—O2	−175.85 (10)
C27—C21—C22—C23	4.8 (2)	C18—N1—Cu—O2	1.49 (12)
N2—C22—C23—C24	175.20 (16)	C22—N2—Cu—O1	176.53 (11)
C21—C22—C23—C24	−6.0 (3)	C28—N2—Cu—O1	−0.34 (12)
C22—C23—C24—C25	0.7 (3)	C22—N2—Cu—O2	−3.48 (11)
C23—C24—C25—C26	3.1 (3)	C28—N2—Cu—O2	179.66 (12)
C24—C25—C26—C27	0.0 (3)	C11—O1—Cu—N1	−4.09 (10)
O2—C21—C27—C26	−177.74 (16)	C11—O1—Cu—N2	171.68 (10)
C22—C21—C27—C26	1.3 (3)	C21—O2—Cu—N1	−179.20 (10)
C25—C26—C27—C21	−4.0 (3)	C21—O2—Cu—N2	5.04 (10)

### Hydrogen-bond geometry (Å, °)

**Table:**

*D*—H···*A*	*D*—H	H···*A*	*D*···*A*	*D*—H···*A*
C18—H18A···O2	0.98	2.47	3.1222 (18)	124
C28—H28A···O1	0.98	2.41	3.0715 (18)	124
